# The Imaging Resolution and Knudsen Effect on the Mass Transport of Shale Gas Assisted by Multi-length Scale X-Ray Computed Tomography

**DOI:** 10.1038/s41598-019-55999-7

**Published:** 2019-12-19

**Authors:** Francesco Iacoviello, Xuekun Lu, Thomas M. Mitchell, Daniel J. L. Brett, Paul R. Shearing

**Affiliations:** 1 University College London, Electrochemical Innovation Lab, Department of Chemical Engineering, London, WC1E 7JE UK; 2 University College London, Department of Earth Sciences, London, WC1E 6BT UK

**Keywords:** Natural gas, Economic geology, Characterization and analytical techniques

## Abstract

The spatial resolution of 3D imaging techniques is often balanced by the achievable field of view. Since pore size in shales spans more than two orders of magnitude, a compromise between representativeness and accuracy of the 3D reconstructed shale microstructure is needed. In this study, we characterise the effect of imaging resolution on the microstructural and mass transport characteristics of shales using micro and nano-computed tomography. 3D mass transport simulation using continuum and numerical physics respectively is also compared to highlight the significance of the Knudsen effect on the reconstructed solid surface. The result shows that porosity measured by micro-CT is 25% lower than nano-CT, resulting in an overestimated pore size distribution and underestimated pore connectivity. This leads to a higher simulated intrinsic permeability. An overestimated diffusive flux and underestimated permeability are obtained from the continuum mass transport simulation compared to the numerical ones when the molecular-wall collision is accounted, evidenced by the large deviation of the measured Knudsen tortuosity factor and permeability correction factor. This study is believed to provide new knowledge in understanding the importance of imaging resolution and gas flow physics on mass transport in porous media.

## Introduction

In recent years shale gas has attracted much attention due to the accessible energy reserves stored in low-permeability organic-rich shales and mudstones. These reservoirs contain a significant amount of hydrocarbons, and the successful exploitation of such resources plays a crucial role in meeting the world’s surging demand for natural gas. This has the potential to play a significant role in the transition to a cleaner energy future due to its high energy content, resulting in lower emissions of carbon and volatile organic compounds (VOCs) at combustion, relative to coal and oil^1^. The gas is released with the help of hydraulic fracturing techniques also known as “fracking”^2^ and gas injection displacement^3^, and is transported through pores of multiple length scales, eventually converging in the main wellbore^4^. While the fracture network greatly determines the productivity of shale reservoirs^5–7^, the transport of shale gas within the matrix also plays an important role^8–10^. Valid pore structure analysis and image-based computational fluid dynamics (CFD) simulation of the shale gas flow in the porous media rely heavily on a faithful 3D representation of the porous microstructure.

Non-destructive three-dimensional X-ray computed tomography (X-ray CT) has been widely applied to the multi-scale microstructure study of the shale gas^7,11^. This technique provides more reliable and representative 3D microstructure compared to those reconstructed by discrete 2D SEM images^12,13^, and helps to mitigate the artefacts of the pore phase potentially introduced from the 2D serial sectioning^14,15^. However, like other imaging techniques, there is a trade-off between the image resolution and the field of view (FOV), and therefore a compromise has to be made between the representativeness and the accuracy of the imaged microstructure, which could inevitably exclude small pores due to the hierarchical pore size distribution in the shale (i.e. ranging from tens of nanometre to micrometre)^16^. A previous study^17^ characterized the gas flow in micro and nanopores using ideal cylindrical pore model, which however cannot account for the effect of complex surface roughness of the wall, the constriction and the arbitrary morphology.

Transport of gas molecules in porous media is mainly governed by two mechanisms: (1) continuum flow, in which the gas molecules interaction is dominant and is often modelled as a viscous effect in continuum physics and (2) the collisions between gas molecules and the wall, also known as molecular flow^18^. The predominant mechanism(s) in the transport regime will depend on the gas species, temperature, pressure and microstructure^19–21^. The Knudsen number *K*_*n*_, calculated as the ratio between the mean free path of the gas molecules and the pore size, is widely used to assess the flow regime in porous media: If *K*_*n*_ < 0.01 (continuum regime), the flow is mainly governed by molecular diffusion and the Knudsen flow can be neglected; if *K*_*n*_ > 10 (Knudsen regime), the gas is highly rarefied and effect of molecular flow outweighs the viscous flow in the continuum regime because of the frequent collisions between the molecules and the porous medium. As for 0.01 < *K*_*n*_ < 10 (transitional regime), shale gas flow is governed by both mechanisms.

The wide distribution of the pore size causes two problems in the mass transport study: (1) it is not reliable to estimate the Knudsen-based diffusivity based on the averaged pore size, which could potentially over-estimate the gas flow due to the constriction effect^21,22^; (2) Viscous flow fails in smaller pore spaces as the diffusion flow mechanisms associated with pore-wall interactions become dominant^23^, which leads to under-estimating the permeability. This means conventional continuum physics can no longer describe the flow field in shales^24^.

To account for the gas molecules-wall interaction (i.e. wall slippage effect), different theoretical models have been adopted to predict the apparent permeability of nanopores, which is a key property for shale gas production. Klinkenberg^25^ analytically addressed the gas-wall collisions by introducing the slippage effect associated with the pressure. Beskok and Karniadakis^26^ mathematically integrated the Knudsen effect into the permeability measurement by comparing the apparent and intrinsic permeability. Tang *et al*.^27^ proved that the apparent permeability is nonlinearly related to the intrisinc permeability.

Direct Simulation Monte Carlo (DSMC) is a numerical method widely used to solve the thermodynamic states of the rarefied gas based on Boltzmann equation, which effectively overcomes the challenges in gas-wall interaction by continuum modelling with conservation equations. Compared to other numerical methods such as molecular dynamics (MD)^3^, DSMC is less computational expensive with high confidence^28^.This method was validated either by experimental permeability^29^ or analytical solution^30^. DSMC has been applied to study the gas flow in a variety of materials of distinct pore morphologies, such as in solid oxide fuel cells^31^, cylindrical channels^32,33^, random/aligned fiber orientations^30,34,35^ and ablative materials^29^.

In this study, we aim to elucidate the effect of imaging resolution on the characterization of porous microstructure and mass transport properties in shales using multi-length scale X-ray CT, followed by the image-based CFD simulation using both continuum and numerical method for the first time to highlight the effect of molecules-wall interaction on the extracted effective mass transport parameters (i.e. Knudsen tortuosity factor, apparent permeability) which could partially be neglected either by mean-field fluid dynamics (continuum flow) or resolution limitation. The particle-based CFD simulation methodology using reconstructed 3D microstructure proposed in this study is highly applicable not only to the shales, but also to be of wide interest across an increasingly broad range of mass transport studies in geological materials.

## Methods

### X-ray Computed Tomography

A cylindrical sample pillar, already employed for previous investigations^7^, was prepared from the shale sample using an A Series/Compact Laser Micromachining System (Oxford Laser, Oxford, UK) following the procedure explained by Bailey *et al*.^36^. The three-dimensional microstructure of shale sample was investigated using two X-ray computed tomography microscopes (Carl Zeiss X-ray Microscopy Inc., Pleasanton, CA): micron-scale Zeiss Xradia 520 Versa (micro-CT) and nano-scale Zeiss Xradia 810 Ultra (nano-CT).

For micro-CT, a total of 1401 radiographs were acquired over a 360° sample rotation range with an exposure time of 35 seconds per radiograph. The shale sample was placed between the X-ray source and a 2k × 2k detector providing a voxel resolution of 224 nm using the 20x objective magnification and a Field of View (FOV) of 448 μm. The instrument was operated at 80 kV. Nano-CT employs post-transmission Fresnel zone plates to achieve resolution in the sub 100 nm range^37^. A total of 1601 projections were collected per 180° sample rotation with an exposure time of 36 seconds. This allowed achieving a set of raw image data with an isotropic voxel resolution of 63 nm and a FOV of 65 μm.

The raw transmission images from both micro-and nano-scale CT imaging experiments were reconstructed using a commercial image reconstruction software package (Zeiss XMReconstructor, Carl Zeiss X-ray Microscopy Inc., Pleasanton, CA), which employs a filtered back-projection algorithm. Tomographic scan details are shown in Table 1. The 3D reconstructed volume of the shale was segmented and analysed using Software Avizo Fire 9.2 (Thermo Fisher Scientific, USA). Due to the low X-ray absorption coefficient difference, it is not possible to distinguish the organic matter (kerogen) from pores based on the reconstructed grayscale data, thus the combined phases are rendered together. This phenomenon is normal in processing X-ray CT data and the same measure was taken in published research^11^. The pore size distribution (PSD) was measured using the plug-in ‘Beat’^38^ in open-source software FiJi^39^.

**Table 1:** Scanning parameters of micro- and nano-CT.

Scan Parameter	micro-CT	nano-CT
X-ray Source energy	80 kV	5.4 keV
Camera binning	1	1
Number of projections	1401	1601
Radiograph exposure time(s)	35	36
Voxel size (nm)	224	63
Field-of-view (FOV, μm)	448	63

### Effective mass transport parameters by continuum fluid dynamics

**Figure 1:**
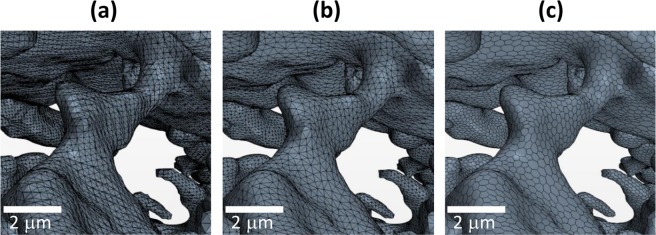
Mesh refinement procedure. (**a**) as-imported triangular surface mesh; (**b**) refined triangular surface mesh; (**c**) generated polyhedral volume mesh.

The surface mesh (ASCII *.stl) file was generated after the segmentation of the porous phase and imported into the commercial computational fluid dynamics (CFD) software Star-CCM+ (CD-Adapco Inc., London). A mesh refinement procedure was undertaken to improve the mesh quality from as-imported raw data (Fig. 1a) to the refined triangular surface mesh (Fig. 1b), to the final polyhedral volume mesh (Fig. 1c). The use of a polyhedral mesh has proven to be more accurate for fluid-flow problems than a hexahedral or tetrahedral mesh of a similar size. The optimized mesh is closed and manifold, with no holes and free edge and the volume change of the porous phase is ensured not to exceed 1% to maintain the microstructural originality.

The tortuosity factor is an effective mass transport parameter representing the effect of complex porous gas pathways on the gas flow^22,40^. In this study, it was measured by CH_4_ ordinary diffusive flow: the CH_4_ molar concentration was set as *c* = 1 mol m^−3^ at the inlet and *c* = 0 at the outlet. It is noted that the tortuosity factor measured by continuum physics is a material parameter and independent of the concentration gradient of the gas. The one-dimensional gas flow *Q*_*e*_ can be described by Fick’s law as

1 $${Q}_{e}=AD\frac{\Delta c}{x}$$

where *D* is the intrinsic diffusivity, *A* is the cross-sectional area of the fluid domain, Δ*c* is the concentration change and *x* is the diffusion length. In the porous medium, Eq. (1) is modified as

2 $${Q}_{p}=\frac{\varepsilon }{\tau }AD\frac{\Delta c}{x}$$

where *τ*_*c*_ is the tortuosity factor, *ε* is the porosity and can be measured by CT data analysis. By dividing *Q*_*e*_ by *Q*_*p*_, the effective transport parameter *ε/τ* can be obtained,

3 $$\frac{{Q}_{p}}{{Q}_{e}}=\frac{\varepsilon }{\tau }$$

It is noted that in the continuum fluid model, the effective transport parameter is independent of the intrinsic diffusivity, indicating that it is a material parameter. The Reynold’s number^41^ of the shale gas flow is far less than unity, which suggests that viscous forces dominate over inertial forces and the permeability can be obtained according to Darcy’s law^42^,

4 $$\frac{\partial P}{\partial x}=-\frac{\mu }{k}v$$

where *k* is the permeability of the porous medium, *v* is the gas velocity, μ is the dynamic viscosity of the gas, *P* is the pressure and *x* is the distance in the flow direction. The intrinsic permeability was obtained by setting a pressure drop (50 Pa) from the inlet to the outlet according to Eq. (4). It is noted that for continuum fluid dynamics, the intrinsic permeability is independent of the pressure gradient.

### Effective mass transport parameters by non-continuum fluid dynamics

To highlight the significance of molecules-wall interactions (Knudsen effect) in the hierarchical porous shale, numerical simulation method Direct Simulation Monte Carlo (DSMC) was used on the 3D reconstructed shale from X-ray CT scan, which is believed to provide a faithful representation of the wall roughness and pore morphology. A sub-volume consisting of 168 × 200 × 200 voxel (10.6 × 12.6 × 12.6 μm) was used in this study.

The Stochastic PArallel Rarefied-gas Time-accurate Analyzer (SPARTA)^43^ DSMC code developed at Sandia National Laboratory (USA) was used in this work. The generated surface mesh (i.e.*stl* file) of the shale was imported into the SPARTA software such that it was embedded in the fluid domain which is composed of an array of 3D Cartesian grids (1.5 million in total). Inter-molecule and molecule-wall collisions were performed following a no-time-counter (NTC) procedure^44^. Shale gas (CH_4_) was simulated from slip flow regime (0.01 < K_n_ ≤ 0.1) to transitional regime (0.1 < K_n_ ≤ 10) with incremental pressure to obtain Knudsen tortuosity factor *τ*_*k*_ based on Eq.(4) and apparent permeability *K*_*a*_ based on Eq. Table as a combination of the ideal gas law, conservation of mass and the differential form of Darcy’s law.,

5 $$J=-\frac{M}{\mu RT}{k}_{a}P\frac{dP}{dx}$$

where *J* denotes the mass flux by DSMC; *M*, *R*, *T*, μ are molecular weight of the gas species, gas constant, the temperature and viscosity respectively. Buffer zones of at least 10% total flow domain were added. A total of 20 million simulation molecules were generated so that the average molecule number in each cell is above 20 to avoid statistical scattering^28^. Each of these simulating molecules is regarded as the representative of a large number of real molecules, the ratio of which is known as scaling factor^45^, to reduce the demand of computational resources. In this study, a scaling factor of 15 was used and small enough to provide accurate DSMC results. The validation of this technique was performed experimentally^31^. The interaction between gas molecules and the porous media can be seen in the video (see Supplementary Video S1).

## Results and Discussion

**Figure 2:**
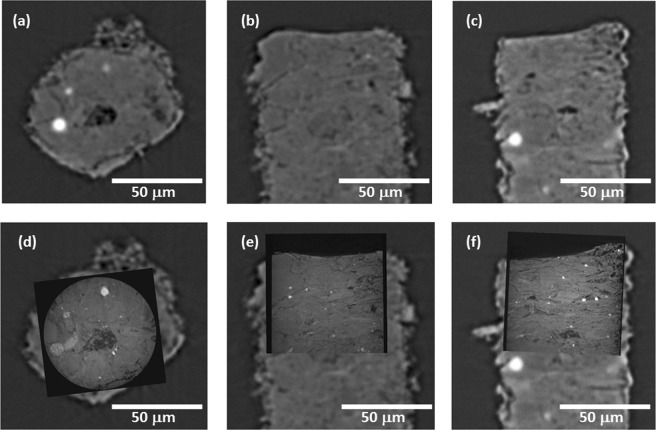
Comparison of the microstructure scanned using micro-CT and nano-CT. (**a**–**c**) micro-CT reconstructed slices of XY, XZ, and YZ views respectively; (**d**–**f**) nano-CT reconstructed slices registered on top of the micro-CT ones, for XY, XZ and YZ views respectively. Scale bar is 50 μm.

The effect of imaging resolution on the reconstructed volume is highlighted in Fig. 2. The top row (a–c) shows the grayscale virtual slices scanned and reconstructed using micro-CT, from which it is clear to see the blurred microstructure due to the resolution limits and it is impossible to extract the pore network with high confidence, particularly for the smallest pores; in the bottom row (d–f), the same region obtained from nano-CT was registered and shown as the superimposition of the micro-CT images. By comparing the obtained microstructures between the top and bottom row, it is found that nano-CT scan provides significantly sharper images so that more microstructural details such as edges and narrow pores which are missing in micro-CT scans can be captured in nano-CT data. In the next section, two case studies will be presented to highlight (1) the effect of imaging resolution on describing the microstructural characteristics and mass transport properties in the direction parallel to the horizontal natural bedding of the shale gas sample; (2) the disparity of obtained mass transport parameters vertical to the natural bedding between continuum and numerical CFD simulation attributed to the captured sub-micron 3D pore network.

### Case study 1: effect of imaging resolution on pore structure and mass transport metrics

**Figure 3:**
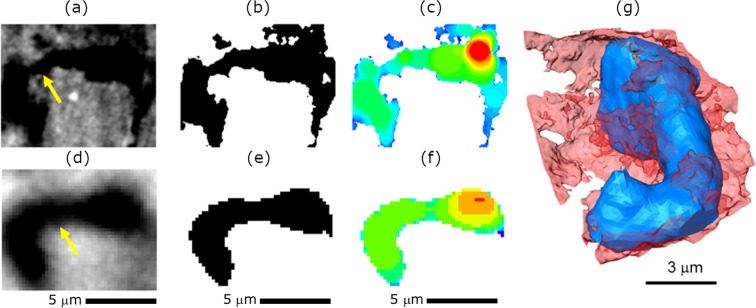
Comparison of the pore microstructure under two resolutions. Figure (**a**–**c**) presents the grayscale, segmented, colour-coded by thickness, skeletonised pore structures obtained based on nano-CT scan; (**d**–**f**) shows the counterparts using micro-CT; (**g**) displays the overlay of the rendered 3D volume of the pore structure under micro-CT (blue) and nano-CT (red).

This study aims to compare the microstructural metrics and mass transport parameters (i.e. tortuosity factor and permeability) as a consequence of the extra porosity which can be imaged in the nano-CT. The same sub-volume was extracted from micro- and nano-CT and compared in Fig. 3. Figure 3a,d compare the morphology of the same pore under two resolutions. The details of the pore edges and curvatures which can be seen in nano-CT (Fig. 3a) are volume-averaged in micro-CT (Fig. 3d). This could lead to an under-segmentation of the pore network from the reconstructed volume (Fig. 3b,e). This local homogenisation effect by micro-CT can result in two disadvantages which undermine further analysis: (1) the pore size distribution and porosity will be over and under-estimated respectively; (2) the percolation will be underestimated as the extracted pore network does not include all of the sub-resolution pores.

After segmentation, a Continuous Pore Size Distribution (C-PSD) analysis was carried out and the PSD is shown as a heatmap with the colour-coded according to its size (Fig. 3c,f). A highly complex pore structure is resolved using nano-CT in contrast to the smoothed single-pore feature using micro-CT. Figure 3g highlights the disparity of the extracted pore structure by overlaying the 3D rendered pore structure under the two resolutions.

**Figure 4:**
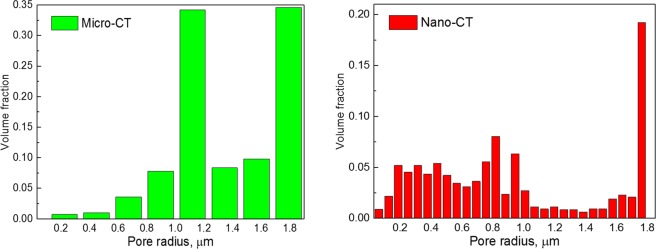
Comparison of the continuous pore size distribution obtained by micro-CT and nano-CT data.

The C-PSD measurement is summarised in Fig. 4. It is observed that with finer features resolved in nano-CT data, the pore size can be quantified with a smaller step size compared to that in micro-CT. Nano-CT scan allows to capture and quantify tinier pores (<1 μm). It is noted that large pores (>1 μm) that are dominant in micro-CT data are not observed in nano-CT measurement. On the other hand, a large amount of pore volume shifts to the low radius end of the histogram. This disparity is speculated as another disadvantage of coarse resolution scan of the shale gas: the complex curvature of the pore edge is homogenised in 3D so that the pore throat resolved in nano-CT (yellow arrow in Fig. 3a) is averaged with the slices above and underneath the plane, resulting in the pore with blurred edge and less dark grey value (Fig. 3d), as a consequence of which, the measured pore size distribution deviates from the practical value.

**Figure 5:**
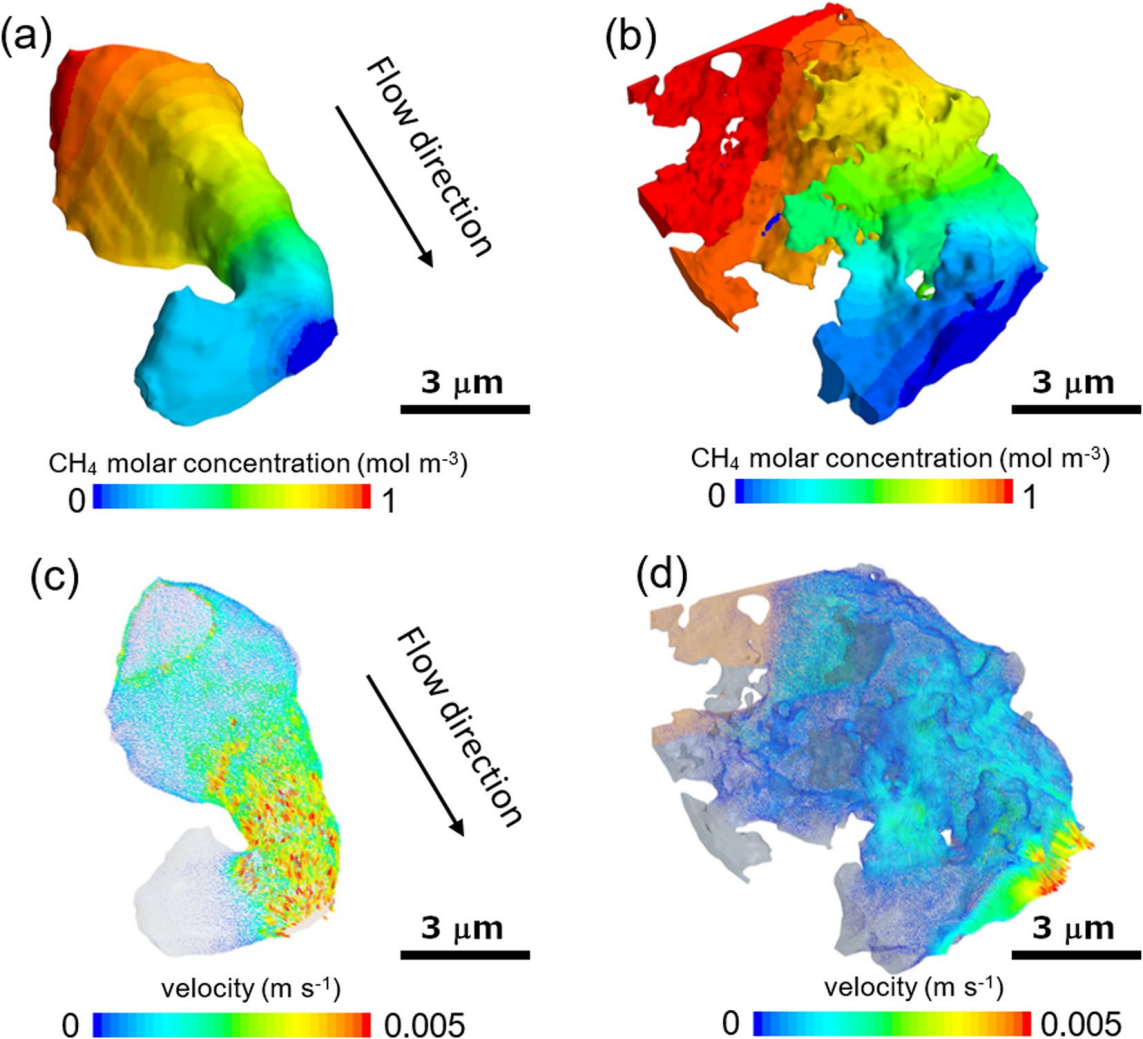
Comparison of the concentration distribution (a and b, micro-CT and nano-CT respectively) and velocity field (c and d, micro-CT and nano-CT respectively) simulated by continuum diffusive and viscous flow to highlight the effect of imaging resolution.

The segmented pores were then meshed and imported to the CFD software Simcenter STAR-CCM+ (Siemens, Plano, TX, USA) for the continuum simulation in order to assess the variation of the measured mass transport properties originating from different imaging resolutions. Figure 5a,b compare the concentration distribution of methane gas at steady state and it is found that for the first half of the flow field the nano-CT pore volume exhibits a much lower concentration gradient (Fig. 5b) compared to micro-CT pore (Fig. 5a), but for the second half of the pore volume the concentration distribution is identical. The reason for this phenomenon is that the nano-CT managed to resolve a larger lateral region of the first half of the pore, providing a higher cross-section for the flow thereby the reduced concentration drop, whilst for the second half more parallel-connected pores are captured in the nano-CT, which in essence would not alter the concentration distribution, instead yielding a higher flow rate. In order words, the morphological difference between two resolution scans mainly consists of laterally-resolved pores which may connect in parallel or in series with the existent pore. However, the velocity field of the viscous flow driven by a constant pressure difference (5 × 10^−4^ bar) is significantly different between the two samples: the micro-CT sample exhibits much higher velocity than the nano-CT one, which is considered as a consequence of the larger surface area and narrower pores in nano-CT, leading to a more remarkable viscous effect. The measured mass transport metrics between the micro-CT and nano-CT samples are summarised in Table 2. The continuum tortuosity factor *τ*_*c*_ between two samples are similar, as is consistent with the concentration field in Fig. 5a,b. The resultant permeability of micro-CT sample is almost one order of magnitude higher than the nano-CT value.

**Table 2:** Summary of the pore structure metrics and mass transport parameters measured by different resolution scans.

Parameter	micro-CT	nano-CT
Porosity (ε)	0.204	0.278
Intrinsic Diffusivity (D (μm))	1.15	0.9
Tortuosity Factor (τ_c_)	1.66	1.8
Permeability (k_c_ (m^2^))	2.08e-14	2.87e-15

### Case study 2: gas flow simulation perpendicular to the bedding direction

This case study aims to investigate the difference of the measured tortuosity factor and permeability when the gas molecules - wall collision is considered in the CFD simulation. This is important as in most of the cases the shale gas flow is governed by transitional and Knudsen regime and thus Darcy flow fails in smaller pore spaces in which the wall-slippage effect becomes dominant. This means the conventional continuum CFD method with the non-slippage condition at the pore-wall interface can no longer faithfully describe the gas flow in the shale. However, few studies have compared the disparity of the extracted tortuosity factor and permeability obtained between continuum and numerical method, and thus the uncertainty is ambiguous. Different from Case Study 1, in which the boundary condition was applied so that the gas flew parallel to the natural bedding direction, Case Study 2 examines the gas transport property vertical to the natural bedding, in which direction the resistance is significantly higher and the gas molecules – wall interaction is more dominant.

**Figure 6:**
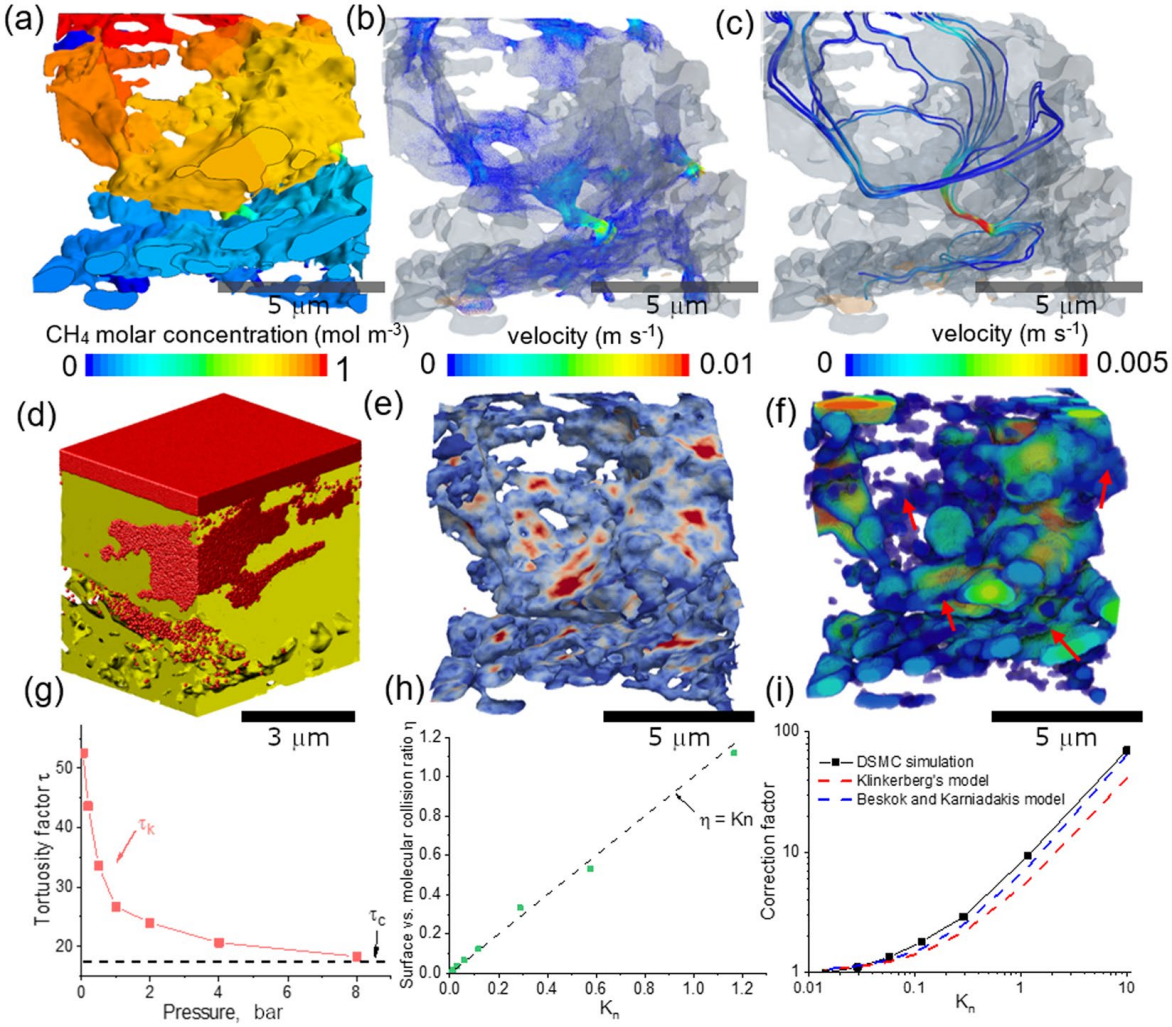
Comparison of the shale gas flow simulation using continuum method and numerical method. (**a**) Concentration distribution of CH_4_ flow perpendicular to the bedding lamination; (**b**) the velocity of the CH_4_; (**c**) streamlines showing the trajectory of gas flow through the narrow pore throat connecting the laminations; (**d**) particle-based CFD simulation with red spheres representing the CH_4_ molecules; (**e**) collision frequency distribution between the gas molecules and the wall; (**f**) the 3D pore size distribution; (**g**) the variation of Knudsen tortuosity factor *τ*_k_ as a function of the gas pressure; (**h**) the ratio of molecules-wall collision and inter-molecules collision as a function of the *K*_*n*_; (**i**) correction factor obtained by DSMC method and compared with the empirical models.

Figure 6a shows the concentration distribution of CH_4_ simulated using continuum CFD method. It is observed that the gradient here is less smooth and uniform compared to Fig. 5a,b, evidenced by a sharp decrease of the concentration at the pore throat vertically connecting the top and bottom half of the horizontally aligned pores. This is the main reason for the strong anisotropy of gas transport in the shale. The narrow pore throat can be visualised in Fig. 6b in terms of the consequent local high velocity of the gas flow. Figure 6c shows some possible streamlines of the gas transport from the top to the bottom, via one of the pore throats. It is noted that the trajectory is highly convoluted and tortuous. The intrinsic permeability measured by the continuum flow is *K*_*i*_ = 7.1 × 10^−19^ m^2^. Figure 6d is a snapshot showing the CH_4_ distribution (red spheres) by particle-based numerical simulation. To the author’s knowledge, this is the first time that DSMC method has been used on the study of shale gas based on the reconstructed 3D volume of the pore network which provides the pore-solid boundary resolved with high confidence at 0.1 μm resolution. This is favourable to examine the collision between the gas molecules and the surface of the wall, as is shown in Fig. 6e. It is observed that the collision does not occur uniformly in the pore volume, instead, it is highly localised at the places where the pore size is smaller than the surrounding areas. This can be supported by comparing with the 3D distribution of the pore diameter Fig. 6f, in which the red arrows point out the corresponding areas of high collision frequency.

Figure 6g compares the Knudsen tortuosity factor *τ*_*k*_ as a function of the gas pressure with that obtained from continuum modelling, which is a constant value independent of the gas pressure. It is found that when the CH_4_ is highly rarefied, *τ*_*k*_ is measured to be as large as 50, then drops drastically with the pressure. At 1 bar, *τ*_*k*_ ≈ 27. The curve asymptotically converges to the continuum tortuosity factor *τ*_*c*_ = 10 when the pressure is above 8 bar, from which and onward continuum flow is dominant. Figure 6g proves that in real-life situations, as the gas pressure is much higher than 8 bar, continuum modelling can be safely applied to the investigated gas shale with the minimal pore size larger than 0.1 μm, which is the imaging resolution of this study. Figure 6h plots the ratio (*η*) of molecules-wall collision and inter-molecules collision as a function of the *K*_*n*_ the two types of collision: molecules-wall and inter-molecules collisions. It is found that the simulated points are linearly proportional to the Knudsen number, aligning with *η* = *K*_*n*_ very well. Finally, a correction factor *f* to the intrinsic permeability *K*_*i*_ as a function of Knudsen number is obtained based on the apparent permeability *K*_*a*_ using DSMC method (*K*_*a*_ = *f*∙*K*_*i*_) (Fig. 6i). When *K*_*n*_ ≤ 0.01, the intrinsic and apparent permeability are in a good agreement, implying the little influence of viscous flow from the wall slippage; with the increase of *K*_*n*_ thus decreasing pressure, the apparent permeability diverges with the intrinsic one, for instance, when *K*_*n*_ = 1, the gas molecules – wall collision is so predominant that the apparent permeability is 9 times larger than the intrinsic one. This result is compared with the empirical solution derived from Klinkerberg’s model^25^ and Beskok/Karniadakis model^26^. Generally, all three curves exhibit the exponential relationship between the correction factor and the Knudsen number, and the measured one by DSMC method is slightly larger than the other two empirical models. This could arise from a variety of factors related to the geometry of the pore structure, such as constriction, shape etc.

## Conclusions

This study firstly compared the difference of reconstructed 3D volume of the shale scanned by X-ray Computed Tomography (CT) using different resolutions (voxel size 224 nm for micro-CT and 63 nm for nano-CT), based on which the continuum CFD simulation was conducted to highlight the effect of imaging resolution on the obtained tortuosity factor and permeability of the shale. The second part of the study discussed the importance of gas molecules-wall collision and wall slippage effect by numerical Direct Monte Carlo Simulation (DSMC) which has been applied to the microstructure-resolved shale model for the first time and then compared the disparity of the mass transport parameters obtained by the conventional continuum CFD modelling. It is found that low-resolution scan has two main disadvantages: (1) the pore size distribution and porosity are over and under-estimated respectively; (2) the percolation is underestimated as the extracted pore network does not include all of the sub-resolution pores. These lead to a much larger intrinsic permeability of the micro-CT scan than the nano-CT one. The morphological difference of the pore structure between two resolution scans mainly consists of laterally-resolved pores which may connect in parallel or in series with the existent pore. The former would not change the concentration distribution but provide a higher mass flow whereas the latter would render a lower local concentration gradient due to the increased cross-sectional flow area. When the surface collision (Knudsen effect) and slippage are considered, the tortuosity factor can be as large as 50 for the most rarefied gas and then significantly drop with the pressure until asymptotically reaching the value (10) obtained by continuum method, implying an over-estimated diffusive flux when the Knudsen effect is not included. In addition, the apparent permeability showed an exponential relationship with the intrinsic one as a function of the Knudsen number, indicating that as the pressure decreases, the deviation of the apparent permeability is larger from the intrinsic one. It is also shown that the ratio of the frequency of the molecular-wall and inter-molecular collision can be estimated by the Knudsen number. As both numerical and continuum simulation methods are widely used in the shale gas study, this study is believed to provides new insights in emphasizing the validity and uncertainty level of the shale gas flow under a variety of conditions. The conclusion drawn can also be used as a reference for the gas flow in other porous media.

## Supplementary information

Supplementary Information

Supplementary Video
