# Supplementary material for: The Imaging Resolution and Knudsen Effect on the Mass Transport of Shale Gas Assisted by Multi-length Scale X-Ray Computed Tomography
Source: Sci Rep. 2019 Dec 19;9:19465. doi: 10.1038/s41598-019-55999-7 (PMC6923360; doi:10.1038/s41598-019-55999-7)
Supplement: Supplementary file 1 — Supplementary Information [file 41598_2019_55999_MOESM1_ESM.docx]

The Imaging Resolution and Knudsen Effect on the Mass Transport of Shale Gas Assisted by Multi-length Scale X-Ray Computed Tomography: Supplementary Information Video S1

Francesco Iacoviello^1^, Xuekun Lu^1, 2^, Thomas M Mitchell^3^, Daniel JL Brett^1^, Paul R Shearing^1^

[1] University College London, Electrochemical Innovation Lab, Department of Chemical Engineering, London, WC1E 7JE, UK

[2] National Physical Laboratory, Hampton Road, Teddington, Middlesex TW11 0LW, UK

[3] University College London, Department of Earth Sciences, London, WC1E 6BT, UK

We submit a video that simulates the gas molecules permeating in the porous media reconstructed by 3D X-ray Computed Tomography. The gas molecules (red) are enlarged for visualisation convenience.
